# A 128-Channel 0.2–8.2 V Calibrated DAC IC Achieving 0.26-LSB DNL and 0.71-LSB DVO for Photonic Computing

**DOI:** 10.3390/mi17091088

**Published:** 2026-09-16

**Authors:** Likai Li, Desong Lv, Jingjing Lv, Dawei Li, Li Du, Yuan Du

**Affiliations:** 1 School of Electronic Science and Engineering, Nanjing University, Nanjing 210023, China; likai_li@smail.nju.edu.cn (L.L.); dslyu@smail.nju.edu.cn (D.L.); ldu@nju.edu.cn (L.D.); 2 School of Microelectronics, Nantong University, Nantong 226019, China; jjlv@ntu.edu.cn; 3 School of Integrated Circuits, Wuhan University of Technology, Wuhan 430070, China; leedavidhust@outlook.com; 4 Interdisciplinary Research Center for Future Intelligent Chips (Chip-X), Nanjing University, Suzhou 215163, China

**Keywords:** photonic computing, thermo-optic phase shifter, R-2R DAC, auxiliary-DAC calibration, DVO

## Abstract

Photonic computing systems require large numbers of accurate programmable voltages for photonic weight programming and device bias control. This paper presents a 128-channel digital-to-analog converter (DAC) implemented in a 250 nm BCD high-voltage CMOS process. A code-dependent per-channel auxiliary-DAC calibration scheme is proposed to compensate main-DAC conversion errors and channel-dependent offsets. In addition, a separated low-/high-voltage-domain driver and a stepwise multichannel update scheme are adopted to reduce static power and suppress update-induced disturbances. After calibration, the measured maximum absolute differential non-linearity (DNL) and integral non-linearity (INL) are 0.26 least significant bit (LSB) and 0.39 LSB, respectively, and the maximum deviation of voltage output (DVO) across 128 channels is 0.71 LSB. The DAC achieves rising/falling slew rates of 6.1/11.7 V/μs under an 8 V output swing. Under dynamic operation with 0.2 to 8.2 V sinusoidal outputs and a 10 kΩ load per channel, the total power consumption is 0.85 W. Thermo-optic phase-shifter measurements further verify programmable photonic phase tuning, demonstrating a scalable electrical control interface for thermo-optic phase-shifter-based photonic computing hardware.

## 1. Introduction

Driven by the rapid growth of artificial-intelligence and data-center workloads, conventional electronic computing is increasingly limited by transistor scaling, memory-access latency, interconnect bandwidth, and energy efficiency [1,2]. Photonic computing offers a promising solution by exploiting the low latency, high bandwidth, and low propagation loss of photonic signal processing [3,4,5,6]. In particular, photonic matrix-vector multiplication can provide massive parallelism and is therefore attractive for next-generation energy-efficient computing systems [7,8].

**Figure 1:**
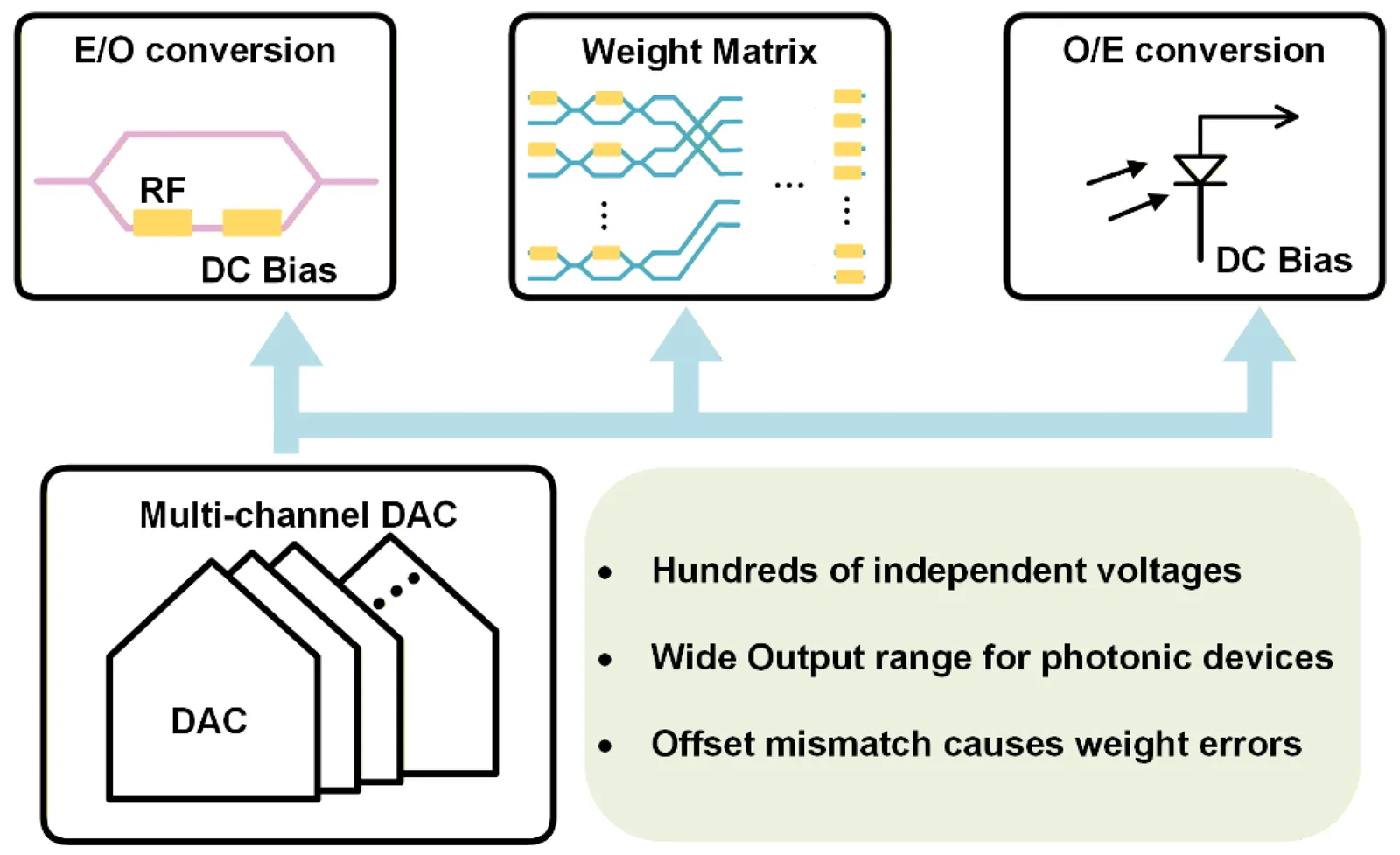
Application scenario of the proposed multichannel DAC for bias and weight control in photonic computing systems.

As shown in Figure 1, in photonic computing systems, key optoelectronic devices, such as modulators, phase shifters, and photodetectors, require stable and accurate analog bias voltages to maintain their optimum operating points. Photonic neural networks further require a large number of programmable analog voltages for weight configuration. For dense MZI-mesh or weight-bank photonic computing architectures without weight sharing, the number of required control voltages typically scales as O(N^2^). Therefore, large-scale multichannel DAC arrays are essential for providing both device bias voltages and programmable weight-control voltages. Such DACs must achieve high channel count, wide output range, good static linearity, and strong channel-to-channel uniformity.

Existing multichannel DAC solutions are not fully suitable for large-scale photonic computing. First, many commercial high-voltage DACs integrate only 16, 32, or 40 channels [9,10], requiring multi-chip cascading for large matrices. For example, a 1024-weight electrical-control interface would require 32 chips when using 32-channel DACs, or 26 chips when using 40-channel DACs. This increases PCB routing complexity, reference distribution difficulty, synchronization overhead, board area, and channel-to-channel mismatch. Second, the output range of many existing multichannel DACs is below 5 V [11,12,13,14], whereas many high-voltage thermo-optic phase shifters and other optoelectronic devices require higher voltage ranges for full-phase tuning. Third, most existing solutions lack native on-chip per-channel offset calibration [15,16]. As a result, channel-dependent offsets must be compensated externally, which increases calibration complexity and limits programmable operation within a finite weight-reconfiguration window.

To address these challenges, this work presents a 128-channel DAC for photonic-computing bias and weight control, implemented in a 250 nm BCD high-voltage CMOS process. Each channel integrates an 8-bit main R-2R DAC, a 4-bit auxiliary DAC, and a programmable-gain high-swing driver, generating programmable dc outputs from 0.2 to 8.2 V after calibration. The main contributions are summarized as follows.

(1) Per-channel auxiliary-DAC calibration: Each channel includes an independent auxiliary DAC that superimposes a fine correction voltage to compensate resistor-ladder mismatch, amplifier offset, and channel-dependent output offsets, thereby improving the voltage uniformity across 128 channels.
(2) Separated low-/high-voltage-domain driver: The front-end small-signal amplifier operates in the low-voltage domain, whereas only the output stage operates in the high-voltage domain. This architecture reduces static power consumption while supporting configurable high-swing outputs.
(3) Low-disturbance multichannel update scheme: Stepwise updating, power-domain isolation, local reference buffering, and layout isolation are employed to suppress disturbances from weight updates to static bias channels, improving output stability when bias and weight control coexist.

The remainder of this paper is organized as follows. Section 2 presents the system architecture and global timing scheme of the proposed 128-channel DAC. Section 3 describes the detailed circuit implementation of the key building blocks. Section 4 presents the measurement setup and silicon measurement results, including mismatch, offset, and output uniformity before and after calibration. Section 5 concludes this work.

## 2. System Architecture

**Figure 2:**
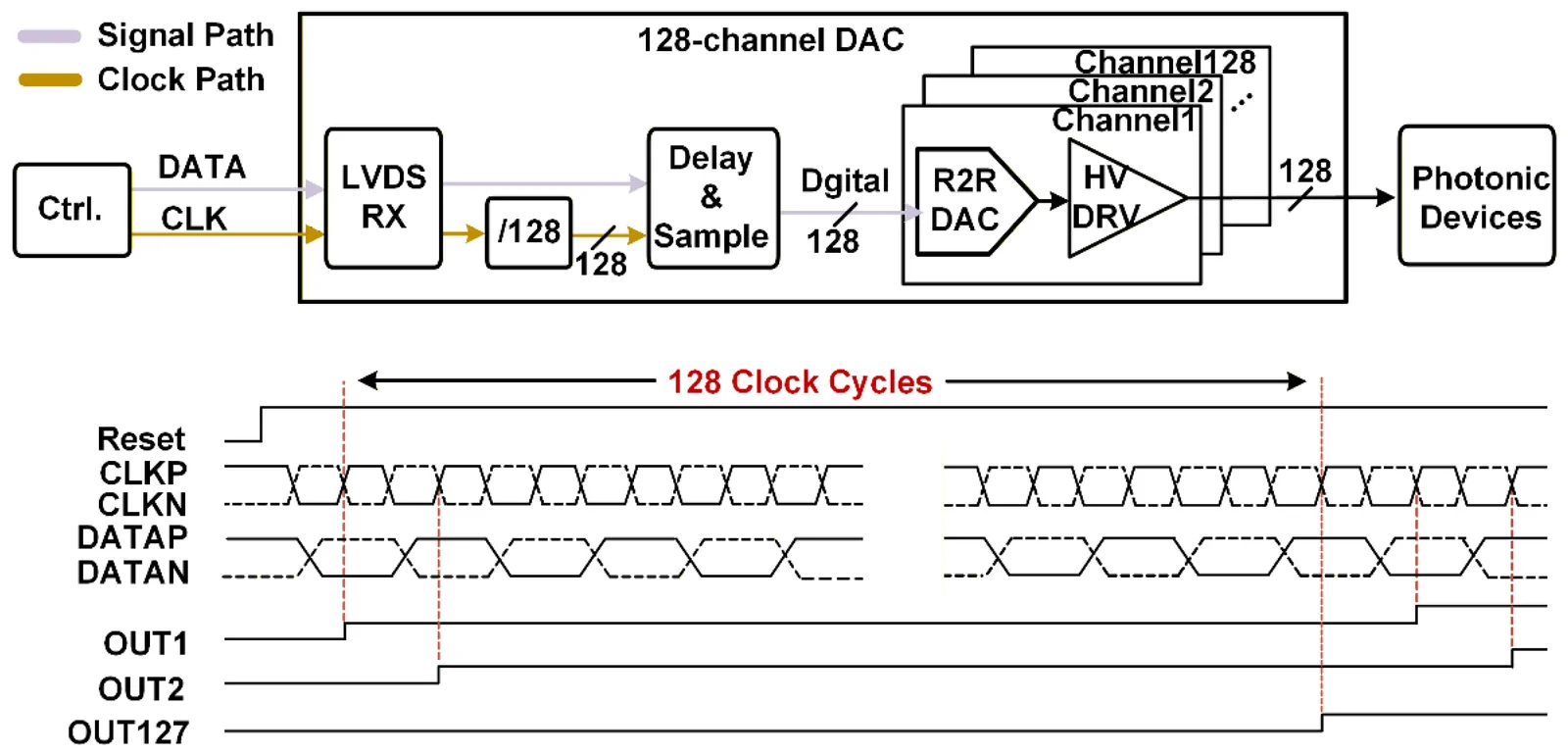
System architecture and global timing scheme of the proposed 128-channel DAC.

Figure 2 shows the top-level architecture and global timing scheme of the proposed 128-channel DAC. The front-end digital interface employs high-speed low-voltage differential signaling (LVDS) differential inputs. The incoming time-multiplexed digital codes and differential clock are first converted by the LVDS receivers into full-swing CMOS signals referenced to the DVDD domain, thereby improving the on-chip digital noise margin. The recovered LVDS clock operates at 128 MHz. It is distributed to a multi-stage synchronous divider and decoding network to generate 128 phase-defined, non-overlapping channel-sampling enables. Therefore, adjacent channels are updated with a time interval of 7.8125 ns, and loading one complete 128-channel frame requires 1 μs. The 8-bit main-DAC code and the corresponding 4-bit auxiliary calibration code are both generated by the external controller. They are transmitted together to the chip and latched into the selected channel register by the same channel-sampling clock. Therefore, the auxiliary DAC is updated synchronously with the main DAC and provides a code-dependent correction rather than a fixed dc trim. These enable the input data to be sequentially latched into 128 independent channel registers, demultiplexing a compact high-speed digital interface into 128 analog output channels and significantly reducing the required pad count.

Each channel adopts a dual-path conversion and summation architecture consisting of a main R-2R DAC and an auxiliary calibration DAC. The main DAC is implemented as an 8-bit R-2R ladder using only two nominal resistor values, R and 2R. Owing to its simple topology, compact area, and low static power consumption, the R-2R DAC is well suited for high-density multichannel integration and generates the nominal analog voltage for weight control. The per-channel auxiliary DAC generates a fine correction voltage according to the calibration strategy and the main-DAC input code. By superimposing this correction voltage in the analog domain, the auxiliary DAC compensates for both intra-channel conversion errors and inter-channel output deviations, thereby improving multichannel output uniformity and static accuracy.

After analog summation of the main-DAC output and the auxiliary calibration voltage, the corrected signal is applied to the back-end high-voltage-compatible driver. The driver separates the low- and high-voltage domains: the front-end small-signal stage operates in the low-voltage domain to reduce power consumption, whereas the output stage operates in the high-voltage domain to provide the required output swing and load-driving capability. In addition, a configurable gain network allows the output range to be adapted to different optoelectronic device requirements, enabling stable driving of on-chip thermo-optic phase shifters, electro-optic modulators, and other optoelectronic loads.

## 3. Circuit Implementation

### 3.1. Auxiliary-DAC-Based Calibration

**Figure 3:**
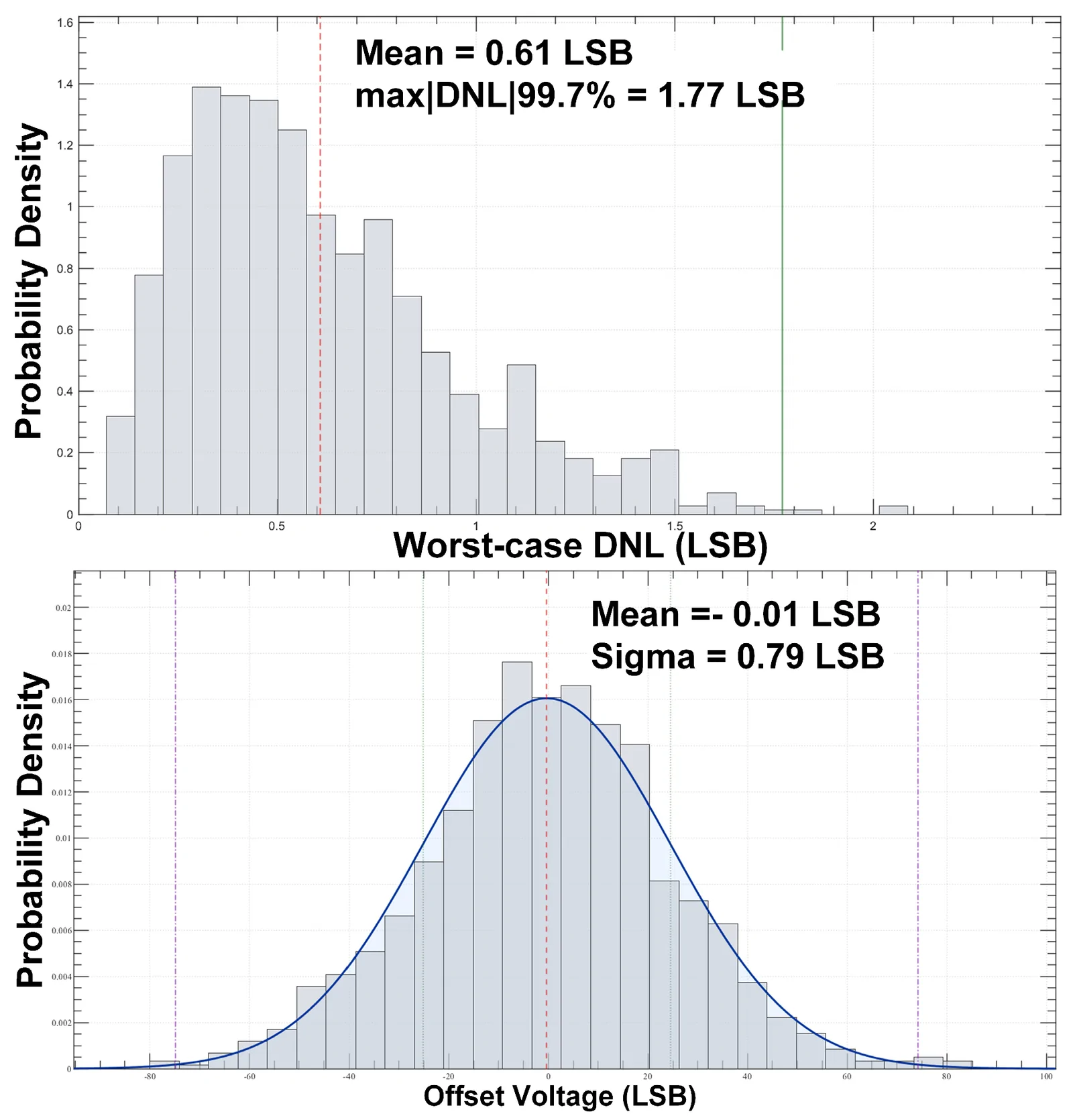
Monte Carlo simulation results of mismatch-induced errors.

The main conversion path adopts an 8-bit R-2R DAC. Compared with a resistor-string DAC, the R-2R architecture features a simple topology, compact area, and good layout repeatability. It is therefore well suited for large-scale multichannel DAC arrays. However, the accuracy of an R-2R DAC relies on the precise binary weighting among different bit branches, rather than uniform voltage division along a resistor string. As a result, resistor-ratio errors, switch on-resistance variations, and layout parasitics directly perturb the actual bit weights. This effect becomes particularly critical during major-code transitions, where the most significant bit (MSB) and higher-order branches dominate the output voltage. Even a small proportional error in these branches can cause the adjacent-code step to deviate from the ideal LSB, degrading DNL and potentially leading to local non-monotonicity under severe mismatch. Therefore, achieving both good static linearity and fast settling using only the main R-2R DAC imposes stringent requirements on resistor matching, resistance selection, and parasitic control. In addition, in a 128-channel DAC array, reference-distribution errors, driver supply/ground drops, amplifier input offset, and feedback-resistor mismatch introduce channel-dependent output errors. Even with the same input code, different channels may produce different output voltages, which directly translate into weight errors or bias-point errors in photonic computing systems. Figure 3 presents the Monte Carlo simulation results of the mismatch-induced intra-channel DNL and inter-channel offset distributions discussed above.

To improve both the code--dependent accuracy of the main DAC and the channel--to--channel output uniformity, an auxiliary DAC is introduced in each channel. Unlike conventional fixed offset trimming, the proposed auxiliary DAC is updated synchronously with the main DAC, and its input code is generated by an external control module (Ctrl.) using a calibration lookup table indexed by both the target channel and the main-DAC input code.

The auxiliary-DAC bit number is determined by the required correction range rather than by the DNL distribution alone. The main DAC, Rmid, and Raux are implemented using the same unit-resistor type and are placed in a matched resistor array; therefore, common process and temperature variations mainly change the absolute resistance values, while their effect on the resistor ratios that determine DNL and INL is expected to be weak. The closed-loop driver gain is also set by the ratio of Rfb and Rin, which are implemented using large-area matched resistor arrays. The remaining process- and temperature-dependent variations mainly affect the required correction range and channel-dependent offset. To verify the correction margin of the selected 4-bit auxiliary DAC, correction-range simulations were performed under SS/TT/FF corners and −40 °C, 27 °C, and 85 °C. The reported value is the correction range required to provide 99.7% coverage over the simulated samples. A total of 1000 Monte Carlo samples were evaluated for each process-and-temperature condition. For each sample, the maximum required auxiliary correction over the complete main-DAC code range was extracted, and the values in Table 1 represent the correction range covering 99.7% of the simulated samples.

The worst required correction range is 6.88 main-DAC LSB. The 4-bit auxiliary DAC provides 16 correction codes, i.e., 15 auxiliary-code intervals. Since each auxiliary step is designed to be approximately 0.5 main-DAC LSB, the available unipolar correction span is approximately 7.5 main-DAC LSB. Therefore, the simulated correction requirement for 99.7% coverage remains within the available auxiliary correction range. No auxiliary-code saturation, missing-code event, or non-monotonic transition is observed in the evaluated cases. This verifies that the 4-bit auxiliary DAC provides sufficient correction range under the simulated mismatch, process, and temperature conditions. For the k-th channel and a given main-DAC code Dk, the Ctrl. provides the corresponding auxiliary-DAC calibration code Qk, and the two codes are written into the target channel simultaneously. The auxiliary DAC therefore serves two purposes. First, it provides point-wise correction for the code-dependent errors of the main R-2R DAC, improving DNL and INL. Second, within the available correction range, it compensates for channel-to-channel output offsets, thereby enhancing the voltage uniformity across 128 channels.

Based on the correction-range analysis summarized in Table 1, a 4-bit auxiliary DAC with 16 unipolar correction levels is adopted. With an effective auxiliary step of approximately 0.5 main-DAC LSB, the available correction range is approximately 0–7.5 LSB, which covers the simulated coverage requirement of 99.7% while maintaining sufficiently fine calibration resolution. The calibrated output voltage can be expressed as

(1) $$\begin{array}{c} V_{dac}=\frac{\left(Vrefh-Vrefl\right)}{255}\sum_{i=1}^{8}2^{i-1}D_{i}+\frac{\left(Vrefh-Vrefl\right)}{255\times 2}\sum_{i=1}^{4}2^{i-1}Q_{i} \end{array}\;\;\begin{array}{c} =(Vrefh-Vrefl)\sum_{i=1}^{8}\frac{2^{i-1}}{255}D_{i}+\frac{\left(Vrefh-Vrefl\right)}{34}\sum_{i=1}^{4}\frac{2^{i-1}}{15}Q_{i} \end{array}\;\begin{array}{c} =V_{main}+\frac{\left(Vrefh-Vrefl\right)}{34(Vrefh\_aux-Vrefl\_aux)}V_{aux} \end{array}$$

**Figure 4:**
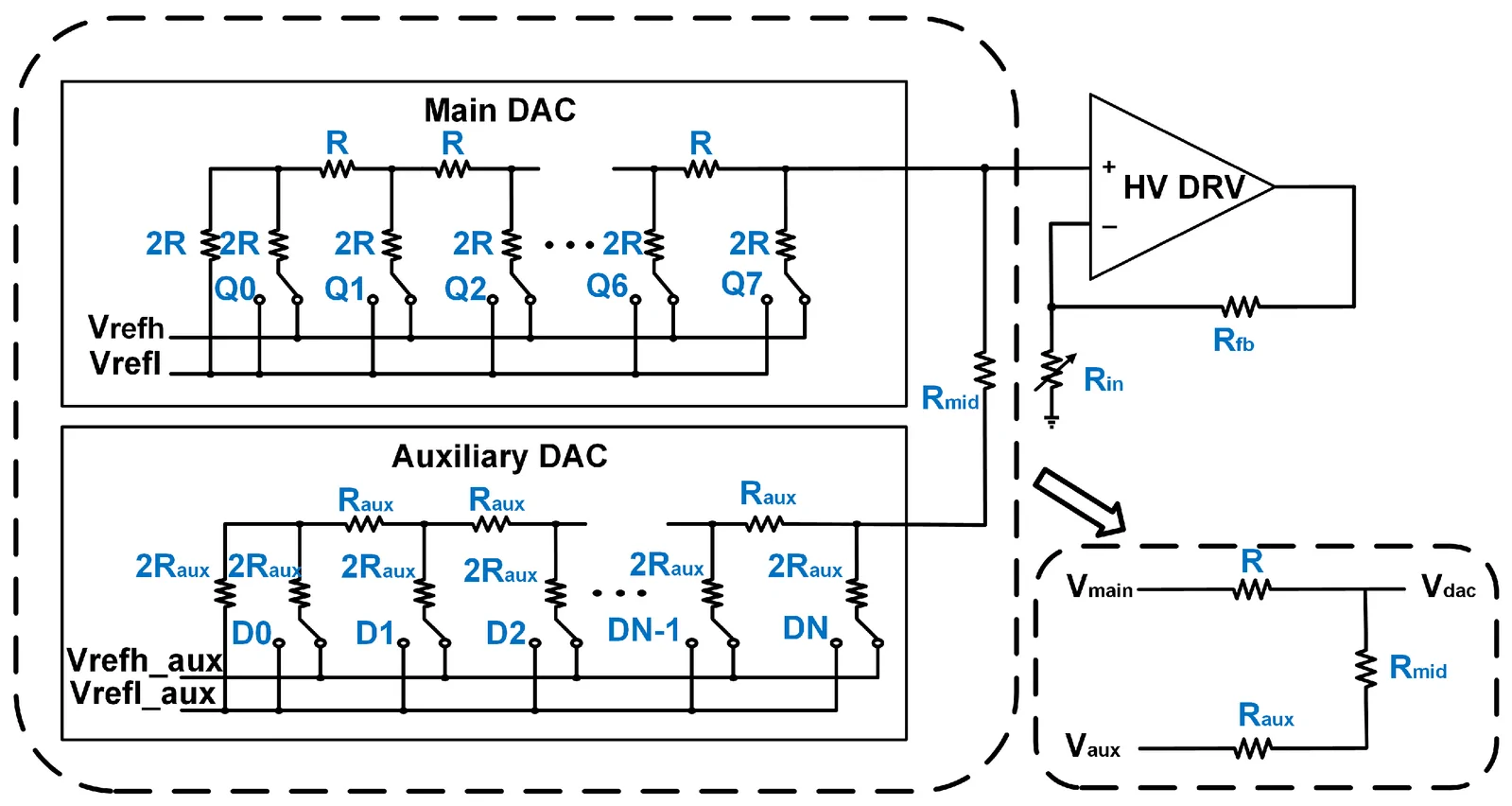
Analog summation calibration scheme using the main DAC and auxiliary DAC.

As shown in the lower-right part of Figure 4, the R-2R DAC output can be modeled by its Thevenin equivalent, consisting of an output voltage Vmain and an output resistance R. When the auxiliary DAC is connected to the analog summing node through Rmid, the single-channel summing voltage becomes

(2) $$V_{dac}=\frac{R_{mid}+R_{aux}}{R+R_{mid}+R_{aux}}V_{main}+\frac{R}{R+R_{mid}+R_{aux}}V_{aux}$$

By setting Rmid = 33R and Raux = R, the summing equation becomes

(3) $$V_{dac}=\frac{34}{35}V_{main}+\frac{1}{35}V_{aux}$$

Therefore, the absolute coefficient of the auxiliary path at the summing node is 1/35, while its coefficient relative to the main path is 1/34. The fixed 34/35 attenuation of the main path is absorbed by the reference setting and the following driver gain. This resistor-ratio selection allows the auxiliary DAC to realize an effective fine-tuning step of approximately 0.5 LSB without requiring an additional low-amplitude reference voltage.

The calibration lookup table is generated experimentally using an external controller and a voltage-measurement setup. A 12-bit 1-MSPS SAR ADC configured for the 0–10 V input range (ADS8638SRGER, Texas Instruments, Dallas, TX, USA; one input channel used) is used to measure the DAC output voltages. The 128 DAC channels are sequentially selected, and all 256 main-DAC codes are applied for each channel. At each code, 20 consecutive samples are acquired and averaged to reduce random measurement noise. Therefore, a total of 128 × 256 = 32,768 channel-code points are measured.

For each channel, the 256 averaged output voltages are used to obtain the channel transfer curve. The code-dependent voltage residual is then converted to the nearest 4-bit auxiliary-DAC code, whose effective step is approximately 0.5 main-DAC LSB. The auxiliary code is constrained to the range of 0–15. Since the auxiliary DAC provides only positive correction, a shifted target curve is used so that the required correction remains non-negative over the calibrated code range. Each main-DAC code receives an independent auxiliary code, and no interpolation between codes is used.

After the per-channel DNL/INL calibration, the channel-to-channel output spread is further reduced by aligning the zero-code outputs of the 128 channels. In this work, DVO means the maximum voltage difference among the 128 channels at the same main-DAC code. The reported maximum DVO is obtained by checking all 256 main-DAC codes and taking the largest channel-to-channel difference. The final maximum DVO over all 256 main-DAC codes is reported in Table 2.

The complete calibration lookup table contains 128 × 256 × 4 = 131,072 bits, corresponding to 16 kB. The auxiliary codes are generated and stored by the external controller or FPGA; the chip itself does not perform auxiliary-code computation. In the present measurement, manual channel switching was used, resulting in a long calibration time. In an automated setup using electronic channel scanning, the ADC conversion time alone is approximately 128 × 256 × 20/1 MSPS = 0.655 s. Including DAC settling, switch settling, and digital-control overhead, the full calibration is expected to be completed on the order of several seconds to tens of seconds.

### 3.2. Low-Power Driver Design

**Figure 5:**
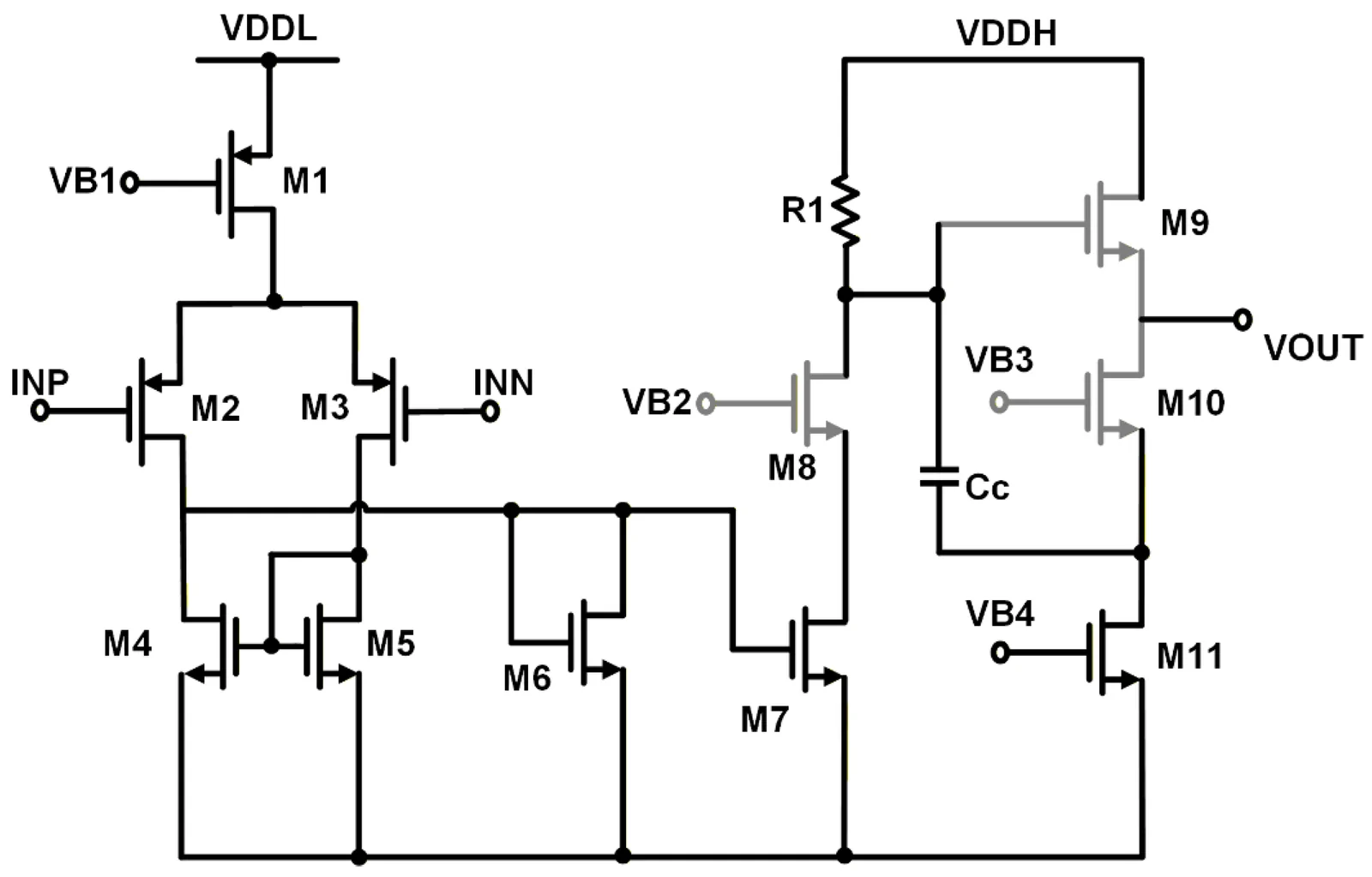
Low-power high-swing driver with separated low- and high-voltage supply domains.

Figure 5 shows the proposed high-swing low-power driver. To reduce static power consumption, a separate low- and high-voltage supply architecture is adopted. The front-end five-transistor operational transconductance amplifier (OTA) operates under the low-voltage supply VDDL, receiving the DAC output and the scaled output-feedback signal from the feedback network for closed-loop error amplification. Since both the DAC output and the feedback comparison node are kept in the low-voltage domain, the front-end small-signal amplifier does not need to be biased directly from the high-voltage supply VDDH. This avoids the additional static power that would otherwise be consumed by a fully high-voltage-biased analog signal chain. The following cascode gain stage and feedforward-compensated source-follower output stage operate under VDDH, providing low-to-high-voltage signal transfer, output swing extension, and load-driving capability. With this low-voltage error amplification and high-voltage output driving architecture, simulation results show that the proposed driver reduces the static power consumption by approximately 25% compared with a conventional fully VDDH-biased driver, while maintaining closed-loop accuracy and output-driving capability.

The front-end five-transistor OTA consists of a differential input pair, current-mirror active load, and tail current source. Owing to its simple topology, low power consumption, and sufficient input sensitivity, it is suitable for low-power error detection between the DAC output and the feedback signal. The intermediate stage employs a cascode structure to increase the effective output resistance and voltage gain, while transferring the control signal from the low-voltage domain to the high-voltage output domain. This stage provides sufficient gate-driving voltage for the output source follower and helps isolate the low-voltage front-end devices from high-voltage nodes, thereby improving circuit reliability under high-voltage operation.

The output stage adopts a feedforward-compensated source-follower topology to enhance the load-driving capability and shorten the output settling time. A conventional source follower biased with a small quiescent current suffers from limited transconductance and slow large-signal charging or discharging of the load capacitance. In the proposed design, a feedforward compensation branch senses fast transitions at the input node through a feedforward capacitor and dynamically modulates the output-stage current during transient events. This mechanism enhances the effective charging and discharging capability during rising and falling transitions, respectively. Since the feedforward path mainly operates during signal transitions, the dynamic driving capability is improved without significantly increasing the quiescent current, resulting in a shorter settling time after driver amplification.

In the measurement, the high-voltage driver supply is VDDH = 13 V, while VDDL, AVDD, and DVDD are all 5 V. The DAC reference voltages are Vrefh = 2.06 V and Vrefl = 0 V. Because the back-end stages operate in the VDDH domain, M8–M10 are implemented using 24 V thick-oxide devices. Transient simulations confirm that the terminal voltages of both the 5 V and 24 V devices remain below the foundry-specified reliability limits under startup, full-swing, and dynamic switching conditions. The 25% static-power reduction is evaluated by comparing the proposed separated-domain driver with a conventional driver in which the entire analog gain chain is biased from VDDH, under the same load, output swing, gain setting, and bias-current condition. Under a 10 kΩ and 5 pF load, the simulated phase margin remains above 53° across the evaluated process and temperature corners.

### 3.3. Supply and Reference Integrity Design

In photonic computing systems, a multichannel DAC is used not only for weight programming of thermo-optic phase-shifter arrays, but also for providing programmable dc bias or reference voltages to electro-optic modulators, photodetectors, and back-end analog front-end circuits. Therefore, during multichannel weight updates, some DAC channels may undergo large code transitions, whereas other channels must maintain stable bias voltages. If the update transients are coupled to static bias channels through the supply, ground, reference network, or substrate, the operating points of the optoelectronic devices can be disturbed. For example, a dc-bias shift in a Mach–Zehnder Modulator (MZM) changes its modulation linear region, while a disturbance in the photodetector reverse bias affects its junction capacitance and response characteristics. These bias errors eventually translate into weight errors or readout errors in photonic multiply-accumulate (MAC) operations.

For a 128-channel DAC array, if all channels are updated simultaneously, the R-2R switching arrays, reference network, and high-voltage driver output stages change states at the same instant. The resulting transient currents are temporally accumulated, causing larger simultaneous switching noise, supply/ground bounce, and reference droop in the on-chip power and reference distribution networks. Although such disturbances can be reduced by increasing the number of reference buffers, strengthening their driving capability, enlarging local decoupling capacitors, or reinforcing the power grid, these approaches introduce additional area and static-power overhead, which is undesirable for scalable multichannel DAC arrays.

Based on the quasi-static nature of weight programming and dc-bias control in photonic computing systems, a deterministic stepwise output-update strategy is adopted in this work. An update interval of Δt is inserted between adjacent channels, so that the DAC switching events and driver output-current transients of different channels are distributed over time. This reduces the peak simultaneous switching current and the transient load on the reference network. In this prototype, the LVDS clock frequency is 128 MHz, corresponding to an update interval of 7.8125 ns between adjacent channels. Loading a complete 128-channel frame therefore requires 1 μs. The complete electrical matrix-update time is determined by the sum of the frame-loading time and the analog settling time following the final channel update. With the measured worst-case settling time of 3.63 μs in the evaluated load range, the complete 128-channel electrical configuration time is below 5 μs. For larger arrays using the same channel-update interval, the loading time scales linearly with channel count, corresponding to 4 μs for 512 channels and 8 μs for 1024 channels, excluding the subsequent analog settling time. It should be noted that the proposed stepwise update does not aim to switch all channel weights at the same instant. Instead, it targets photonic computing modes that allow a finite weight-reconfiguration window. In this mode, the key requirements are that the complete weight matrix settles to the target state within the specified configuration time and that the disturbance on inactive or static bias channels remains sufficiently small during the update process.

In addition, supply- and reference-integrity-aware implementation techniques are employed in the layout and power distribution. The digital supply/ground, low-voltage analog supply/ground, and high-voltage driver supply/ground are routed in separated domains to reduce coupling from digital switching and high-voltage load transients to sensitive analog nodes. Guard rings are inserted around critical analog blocks and high-voltage drivers for substrate isolation. Center power pads and wide metal supply routes are also used to reduce the on-chip supply-path impedance.

**Figure 6:**
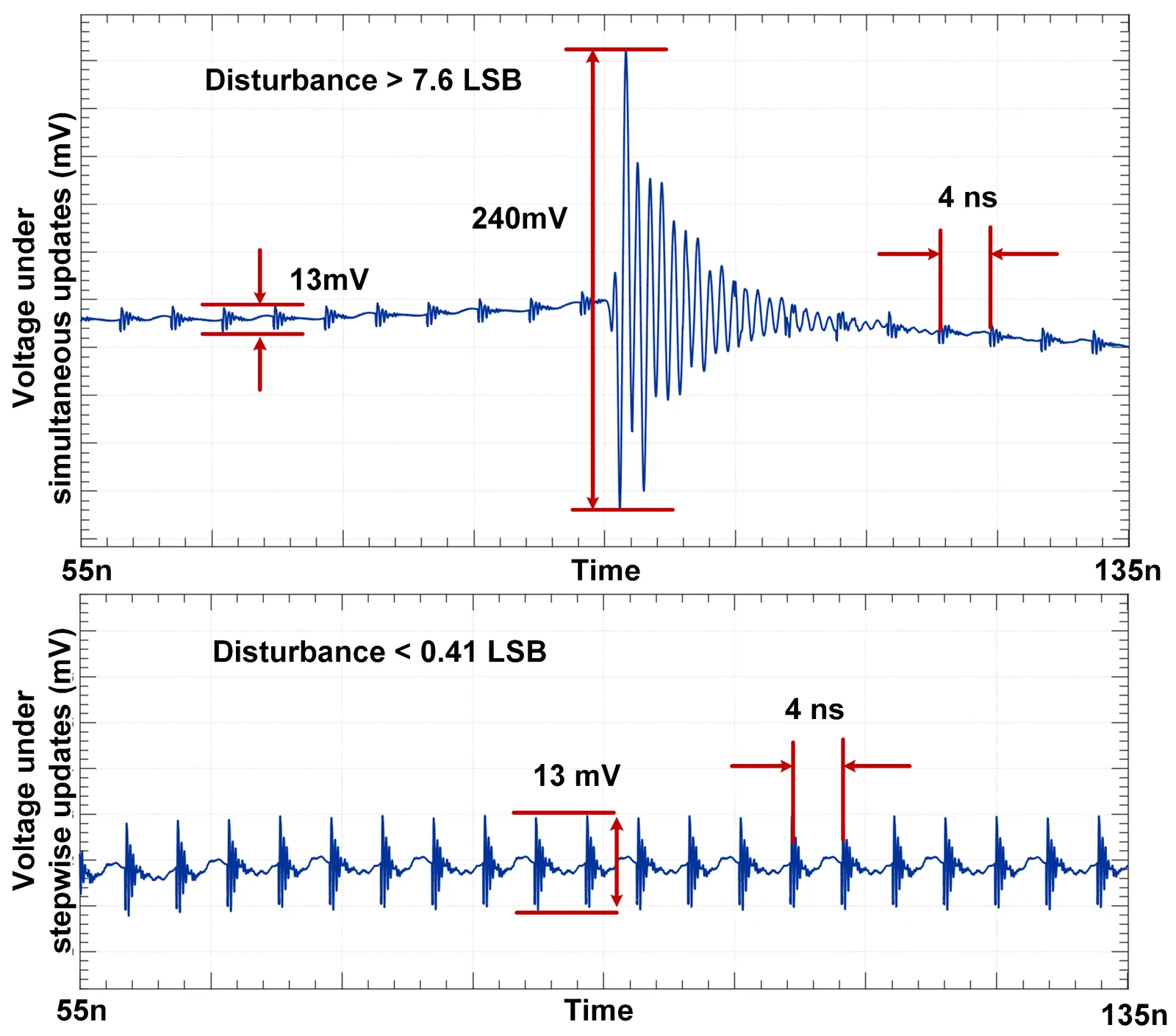
Transient simulation comparison of static-channel disturbance under simultaneous and stepwise updates.

As shown in Figure 6, transient simulations were performed to compare simultaneous and stepwise update schemes. Under the same input codes, reference voltages, and load conditions, simultaneous updating causes concentrated multichannel switching transients, resulting in larger disturbances at both output nodes and static bias nodes. In contrast, the stepwise update spreads the transient events over time, reducing both the static-channel output disturbance and the reference-voltage fluctuation. Simulation results show that, with the proposed stepwise update, the maximum disturbance of a static channel is limited to 13 mV, corresponding to approximately 0.41 LSB. In silicon measurement, the update-related waveform fluctuation is not distinguishable from the single-shot output fluctuation of approximately 22 mVpp under the present measurement bandwidth. Therefore, the proposed stepwise update suppresses the static-channel disturbance below the measured single-shot waveform fluctuation, while avoiding excessive reference-buffer oversizing.

## 4. Results

**Figure 7:**
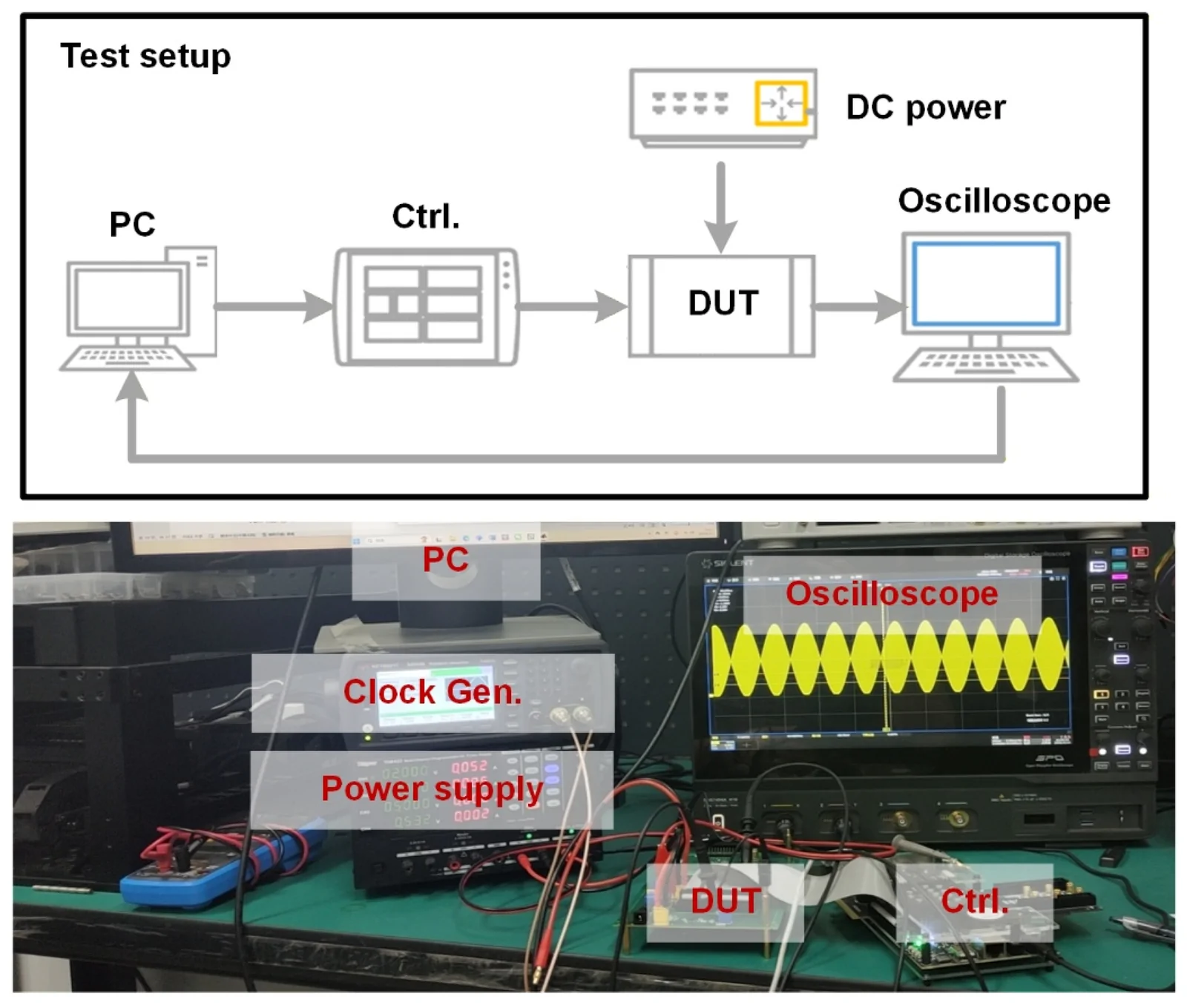
Test environment and system setup.

Figure 7 shows the measurement setup of the proposed 128-channel digital-to-analog converter (DAC). The test platform consists of an external control module, a customized printed circuit board (PCB), external power supplies, an oscilloscope, and a data post-processing program. The control module provides the digital input codes and clock signals to the chip under test, and controls channel selection, data loading, and calibration-code updating according to the predefined timing sequence. A 13 V external supply is applied to the PCB and converted by on-board dc–dc converters and low-dropout regulators (LDOs) to generate the required digital supply, low-voltage analog supply, high-voltage driver supply, and reference voltages. The DAC outputs are routed from the test board to the oscilloscope for waveform acquisition. The measured voltage data are then post-processed to extract key performance metrics, including DNL, INL, inter-channel offset, transient response time, and slew rate.

**Figure 8:**
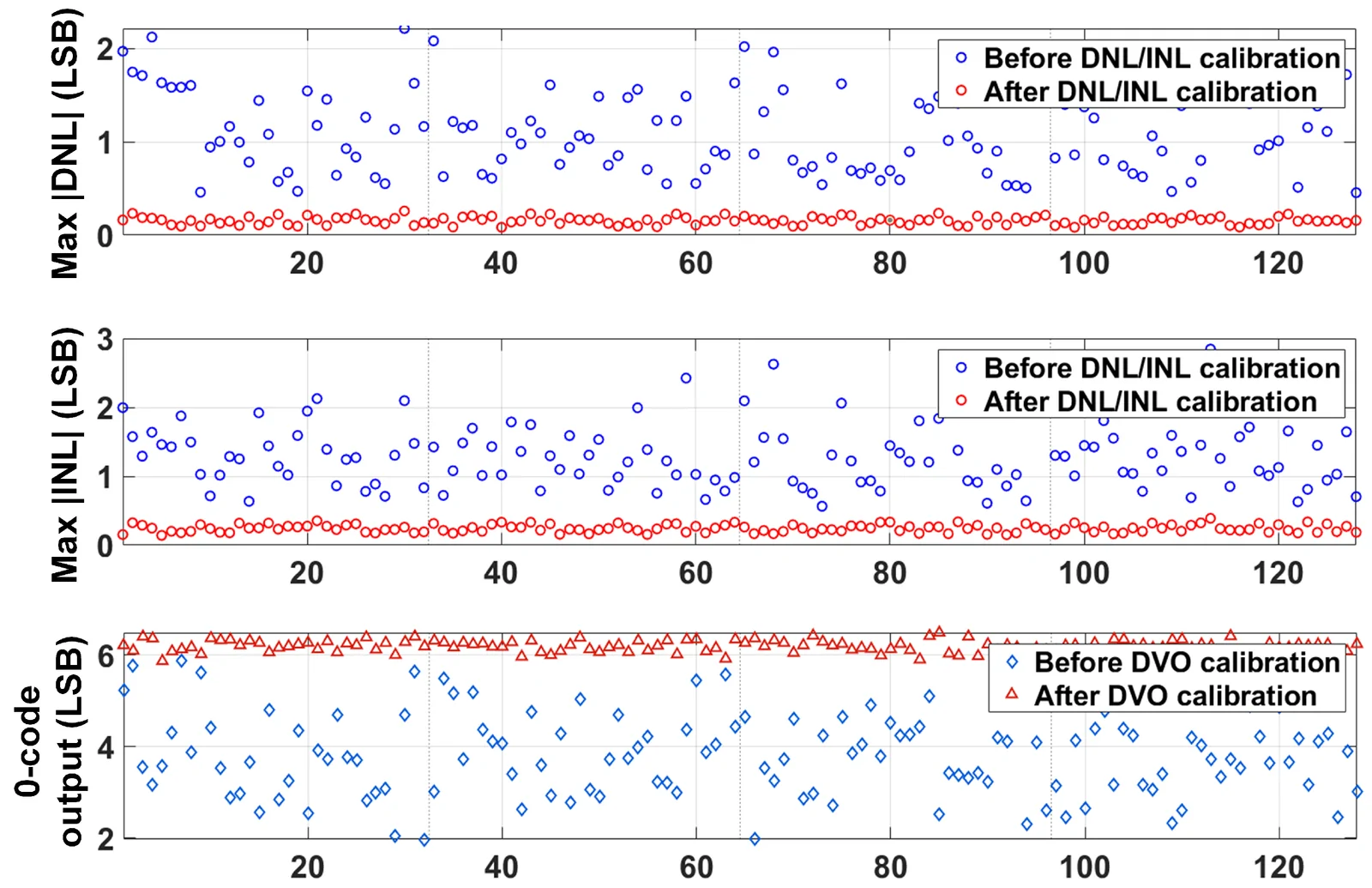
Static calibration results of a representative measured die in physical channel order. From top to bottom, the three panels show the maximum absolute DNL per channel, the maximum absolute INL per channel, and the zero-code output distribution of all 128 channels before and after calibration. The lower panel illustrates the zero-code DVO-alignment effect; the maximum global DVO over all 256 codes is reported in Table 2.

The static performance of the chip is first characterized. Figure 8 presents the calibration results of a representative measured die in physical channel order. The upper and middle panels show the maximum absolute DNL and INL, respectively, for each of the 128 channels before and after the code-dependent DNL/INL calibration. The worst post-calibration DNL and INL are reduced to 0.26 LSB and 0.39 LSB, respectively. The lower panel shows the zero-code output distribution before and after the channel-dependent DVO-alignment step. The zero-code channel spread is reduced from 3.887 LSB to 0.620 LSB. This lower panel illustrates the effect of the zero-code alignment; it does not directly represent the maximum global DVO. The maximum global DVO is evaluated over all 256 main-DAC codes and is reported for each die in Table 2.

**Figure 9:**
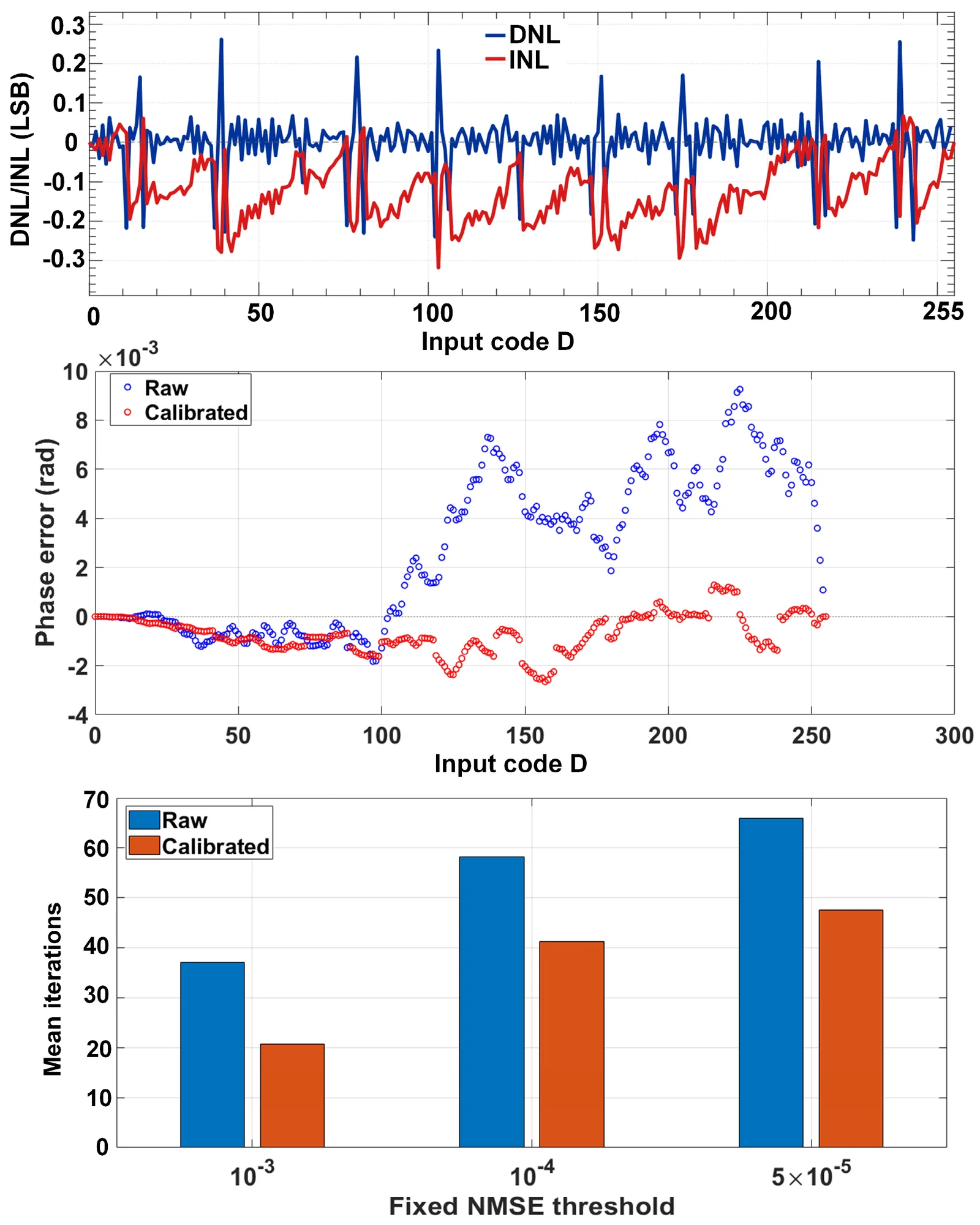
Post-calibration static linearity and photonic error projection. From top to bottom, the panels show the post-calibration DNL/INL curves of a representative channel, the phase error versus main-DAC code before and after calibration, and the black-box gradient-descent training iterations required to reach fixed NMSE thresholds using measured DAC-induced errors.

Figure 9 further shows the post-calibration code-by-code static linearity and its impact on photonic phase and weight programming. The first panel shows the post-calibration DNL and INL curves of a representative channel. The residual DNL peaks and the corresponding INL periodicity are mainly associated with residual R-2R bit-weight mismatch at major carry transitions. In addition, the discrete 4-bit auxiliary correction code can introduce small local discontinuities when the auxiliary code changes between adjacent main-DAC codes. After calibration, these residual errors are suppressed within the reported DNL/INL limits.

The thermo-optic phase shifter used for optical validation is fabricated in a 130 nm silicon-photonics process. The heater resistance is approximately 10 kΩ, and the phase-shifter length is 200 μm. During measurement, the optical output is converted to the electrical domain using a photodetector and a transimpedance amplifier. The phase response is then extracted from the measured electrical signal and fitted as a function of the squared DAC output voltage, which is consistent with Joule heating in a resistive thermo-optic tuner. The extracted tuning efficiency is approximately 2.88 mW/π. The second panel of Figure 9 shows the phase error versus main-DAC code before and after calibration. The calibration significantly suppresses the phase-error variation over the control range. To further quantify the impact on photonic weight programming, the complex phase weight is modeled using the extracted phase response. The normalized weight NMSE is reduced from 2.1098 × 10^−5^ before calibration to 6.5499 × 10^−7^ after calibration, corresponding to a 32.2-fold reduction.

The training-iteration plot in Figure 9 presents a black-box gradient-descent training using the measured DAC-induced errors. Fewer iterations are required after calibration to reach the same fixed NMSE thresholds.

Table 2 summarizes the measured static performance of three fabricated dies. Across the three dies, the worst post-calibration DNL, INL, and global DVO are 0.26 LSB, 0.39 LSB, and 0.71 LSB, respectively. The channels associated with the worst DNL, INL, and DVO results are channels 30, 113, and 65, respectively. These channels are basically located close to the die edge or corner, suggesting that layout-gradient and edge effects may contribute to the residual spread.

**Figure 10:**
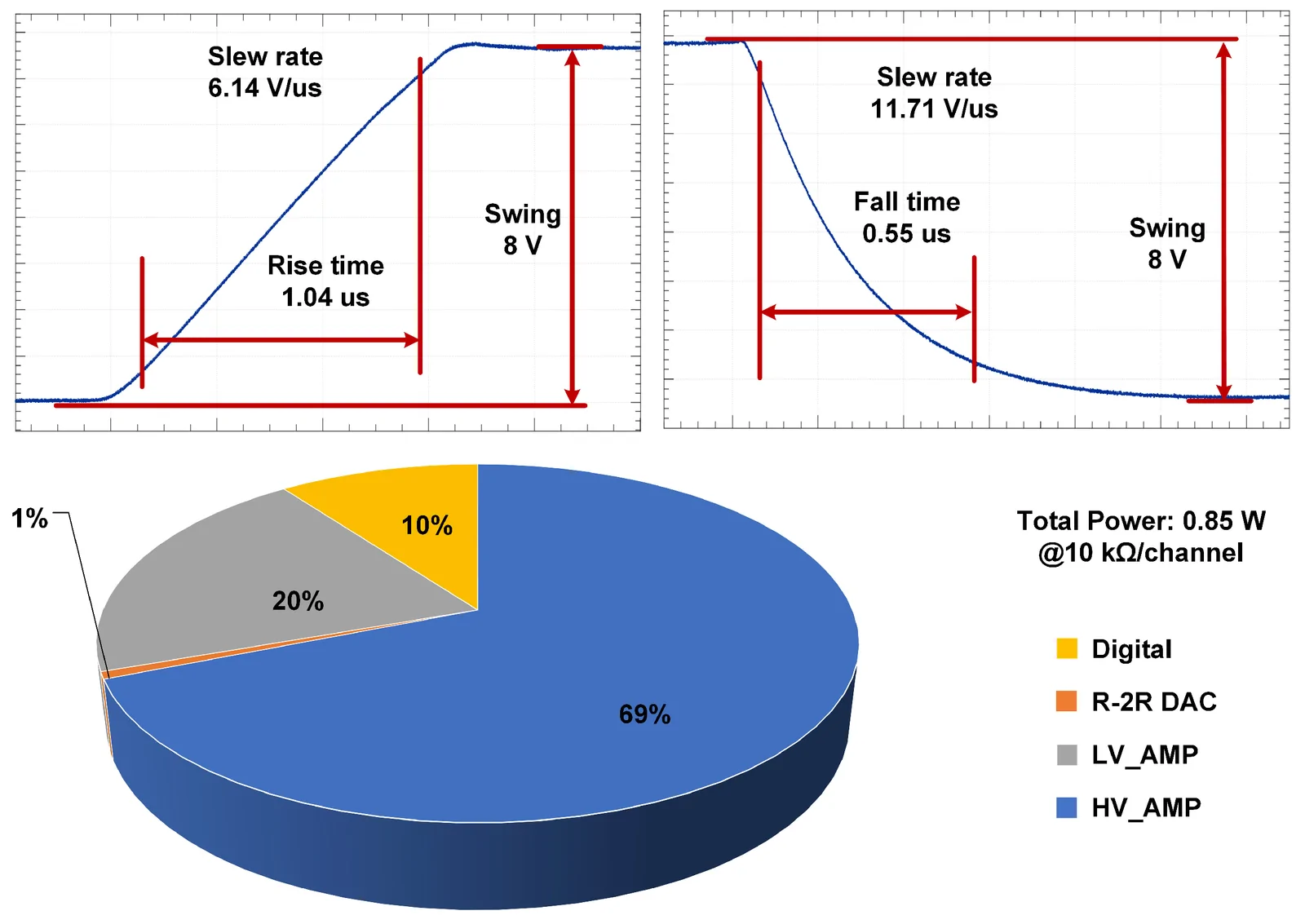
Measured large-signal transient response and power consumption under an 8 V output swing.

The dynamic performance is then evaluated. Figure 10 shows the large-signal transient response and power analysis of the driver under an 8 V output swing. The measured 10–90% rising transition time is 1.04 μs, and the 90–10% falling transition time is 0.55 μs. The corresponding slew rate is obtained from the effective voltage transition under the 8 V output swing. These results indicate that the proposed separated-domain high-voltage driver provides sufficient dynamic driving capability over the 0.2 to 8.2 V output range. As observed from the measured waveforms, the update-induced disturbance is almost buried in the noise floor during both rising and falling transitions, indicating that the update-related perturbation is below the single-shot waveform fluctuation in the present measurement setup. For power characterization, a 0.2-to-8.2 V sinusoidal waveform is generated at the DAC output, and the average current of each supply domain is measured. The reported value therefore represents the DAC-chip supply power under external-load-driving operation. It excludes the external FPGA/controller, measurement instruments, and the conversion losses of the board-level DC-DC converters and LDOs. The total IC supply power is approximately 0.85 W under the dynamic operating condition with a 10 kΩ load per channel. The measured power breakdown is 85.6 mW for digital logic, 5.4 mW for the R-2R and auxiliary DAC array reference supply, 171.6 mW for the low-voltage amplifier, and 582.4 mW for the high-voltage driver under the sinusoidal-output condition. Therefore, the high-voltage driver and load-driving path dominate the total power.

Table 3 summarizes the measured rising and falling settling times under different resistive and capacitive loads. The settling time is extracted from the 50% crossing of the selected channel-sampling clock edge to the time when the output enters and remains within the ±0.5-LSB error band around the final value. Since one nominal LSB is 8 V/255 = 31.37 mV, the ±0.5-LSB error band corresponds to ±15.69 mV.

The listed values are rise/fall settling times. The parasitic-only condition includes oscilloscope-probe and package parasitics without an additional external capacitor. The rising settling time remains nearly constant at approximately 1.83 μs across the evaluated load range, indicating that the rising transition is mainly limited by the internal gate-driving node or closed-loop response rather than by the output capacitive loading. In contrast, the falling settling time increases from 0.93 μs to 3.63 μs as RL increases and CL increases. This is consistent with the asymmetric source-follower output stage: during rising transitions, the output device actively charges the load, whereas during falling transitions, the output capacitance is discharged through the pull-down path and the resistive load. The 10–90% falling slew rate decreases from 12.2 V/μs under 5 kΩ with parasitic-only capacitance to 5.2 V/μs under 15 kΩ with 10 pF, while the rising slew rate remains approximately 6.1 V/μs.

To evaluate time-domain output stability, all 128 channels were held at the full-scale output code for three hours under laboratory ambient conditions without temperature-chamber control. The common output drift of the 128-channel array is approximately 12 mV over three hours, corresponding to 0.38 LSB. During the measurement, the maximum channel-to-channel deviation remains within 0.8 LSB. Since no controlled temperature sweep was performed, this result is reported as a three-hour DC drift measurement rather than a temperature-drift characterization.

**Figure 11:**
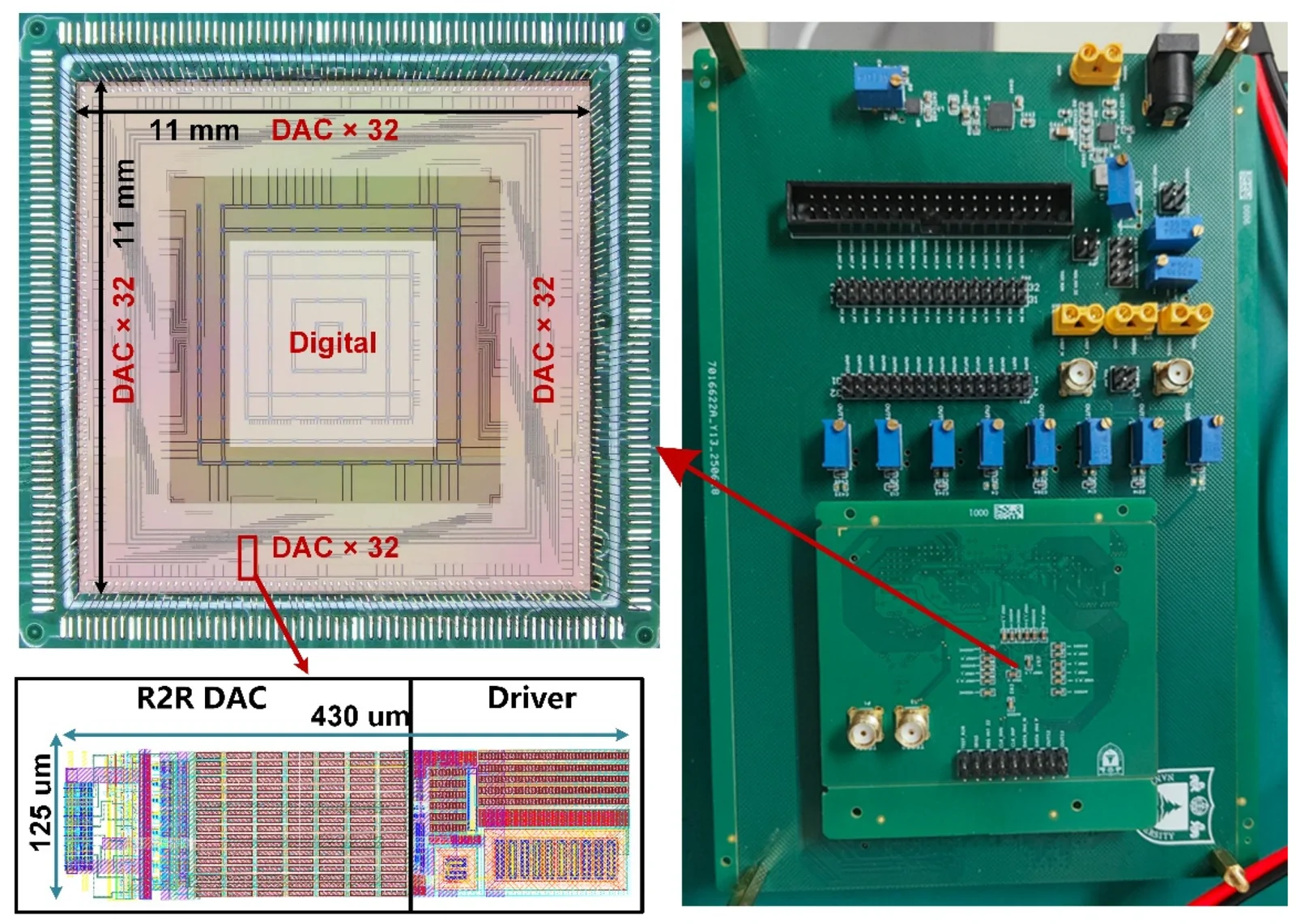
Die photo, single-channel layout, and test PCB of the proposed 128-channel DAC.

Figure 11 shows the chip micrograph, single-channel layout, and test PCB. The 128 output channels are arranged along the four sides of the chip, with 32 channels placed on each side. Center power pads are inserted to reduce the supply-path impedance of the high-voltage output stages and to improve supply integrity during multichannel operation. This pad arrangement is compatible with both wire-bonding and flip-chip packaging, providing flexibility for future system-level integration. The right side of Figure 11 shows the test PCB. The chip is mounted on the backside of the daughter board, which is connected to the motherboard through FPGA mezzanine card (FMC) connectors. The motherboard provides digital data, clocks, supplies, and bias voltages to the daughter board, while the DAC outputs are routed back to the motherboard through the FMC interface for measurement. Table 4 summarizes the performance comparison between this work and previously reported multichannel DAC.

The die size is 11 mm × 11 mm, corresponding to 121 mm^2^. The active single-channel area is 0.05375 mm^2^, including 0.0325 mm^2^ for the main R-2R DAC, Rmid, and auxiliary resistor array, and 0.02125 mm^2^ for the high-voltage driver. The signal pads occupy approximately 2.43 mm^2^, and the internal flip-chip power pads occupy approximately 1.33 mm^2^. The adopted 250 nm BCD PDK does not support placing signal pads directly over the active circuit area; therefore, the signal pads are arranged along the four sides of the die. The DAC channels are placed close to the peripheral output pads to reduce output-routing parasitics, while the central region is used for power pads, digital decoding, clock distribution, and tree-structured timing routes. Future implementations using pad-over-active-area or flip-chip-compatible routing could further reduce the die footprint and improve supply and thermal distribution.

## 5. Conclusions and Discussion

This work presents a 128-channel high-voltage DAC for photonic-computing bias and weight control. Implemented in a 250 nm BCD CMOS process, the proposed chip provides programmable 0.2 to 8.2 V calibrated outputs for driving thermo-optic phase shifters, electro-optic modulators, photodetector bias nodes, and other optoelectronic loads. Each channel integrates an 8-bit R-2R main DAC, a 4-bit auxiliary calibration DAC, and a programmable-gain high-voltage driver. The main DAC generates the nominal weight or bias voltage, while the auxiliary DAC provides code-dependent fine correction to compensate main-DAC conversion errors and channel-dependent offsets. Measurements across three fabricated dies show worst-case post-calibration DNL, INL, and global DVO values of 0.26 LSB, 0.39 LSB, and 0.71 LSB, respectively. The calibrated photonic-weight NMSE is reduced by 32.2 times, while the complete 128-channel electrical configuration time remains below 5 μs under the evaluated load conditions.

The measurement results also validate the design hypotheses of this work. First, the auxiliary-DAC calibration confirms that the dominant static errors in a dense R-2R DAC array can be effectively reduced by local analog correction rather than by relying solely on resistor matching. This is particularly important for large photonic weight matrices, where channel-dependent voltage errors directly translate into photonic weight errors. Second, the separated low-/high-voltage-domain driver verifies that high-voltage output swing can be supported without forcing the entire analog signal chain to operate from the high-voltage supply. The measured 8 V output swing and rising/falling slew rates of 6.1/11.7 V/μs demonstrate that the proposed driver can provide sufficient dynamic capability for programmable optoelectronic-device control. Third, the thermo-optic phase-shifter measurement confirms that the DAC output can be used not only as an electrical voltage source but also as a practical photonic phase-control interface.

Several aspects require further optimization when scaling this architecture to larger photonic computing systems. The first issue is power consumption. In this prototype, the measured total power is largely affected by the 10 kΩ/channel resistive load used to emulate or drive thermo-optic phase shifters. For large arrays, the load power of resistive phase shifters can dominate the overall system power. From the circuit perspective, future drivers should explore more efficient high-voltage output stages, adaptive biasing, output-stage power gating, and activity-aware update schemes to reduce static and dynamic overhead. From the optoelectronic-device perspective, improving phase-shifter efficiency or reducing the required tuning voltage would directly reduce the output swing and current demand on the DAC.

The second issue is area scaling. Although the R-2R main DAC and auxiliary DAC provide a compact and repeatable channel structure, simply shrinking the channel pitch is not sufficient for larger arrays. Electrical crosstalk, reference coupling, supply impedance, substrate noise, and thermal density must be considered together. In particular, high-voltage output stages and resistive optoelectronic loads may generate non-negligible heat, which can disturb neighboring optoelectronic devices and degrade long-term output stability. Future implementations should therefore co-optimize the channel pitch, power-grid density, reference distribution, guard-ring isolation, and thermal spreading paths. Flip-chip packaging and distributed power pads may further reduce supply impedance and improve heat removal in larger-channel-count arrays.

The third issue is calibration scalability. The proposed code-dependent auxiliary-DAC calibration improves accuracy, but the amount of calibration data increases with both channel count and DAC resolution. For future arrays with hundreds or thousands of channels, storing a full calibration table for every channel and every code may become inefficient. Since this work focuses on circuit implementation, future research may investigate circuit-friendly calibration compression methods, such as segment-wise calibration, major-code calibration, interpolation between stored calibration points, or shared correction parameters for physically matched channel groups. These approaches can reduce memory overhead while preserving most of the static-accuracy improvement provided by auxiliary-DAC correction.

Overall, the proposed 128-channel DAC demonstrates a scalable circuit framework for high-voltage, high-uniformity, and calibrated voltage generation in photonic computing systems. Further improvements in driver efficiency, thermal-aware layout, reference integrity, packaging, and calibration-memory scaling will be essential for extending this approach toward larger photonic weight matrices and more densely integrated photonic computing platforms.

## Figures and Tables

**Table 1:** Simulated auxiliary correction requirement for 99.7% coverage under process and temperature conditions.

Required Correction Range (Main-DAC LSB)	SS	TT	FF
−40 °C	6.88	6.67	6.56
27 °C	6.66	6.62	6.57
85 °C	6.36	6.44	6.27

**Table 2:** Three-die static performance summary before and after calibration.

Die	Raw DNL (LSB)	Raw INL (LSB)	Raw DVO (LSB)	Cal. DNL (LSB)	Cal. INL (LSB)	Cal. DVO (LSB)	Gain Error %
1	2.06	2.41	4.43	0.23	0.34	0.61	0.01
2	2.21	2.38	4.22	0.26	0.39	0.66	0.01
3	2.21	2.85	3.92	0.24	0.36	0.71	0.01

**Table 3:** Measured settling time under different load conditions.

Settling Time (Rise/Fall)	5 kΩ	10 kΩ	15 kΩ
**Parasitic only**	1.83 μs/0.93 μs	1.83 μs/1.68 μs	1.83 μs/2.38 μs
**5 pF**	1.83 μs/1.21 μs	1.83 μs/2.34 μs	1.84 μs/2.98 μs
**10 pF**	1.84 μs/1.58 μs	1.83 μs/2.78 μs	1.83 μs/3.63 μs

**Table 4:** Comparison with the state of the art.

Reference	AD5371 [9]	VLSI 23 [16]	ASSCC 23 [12]	ISSCC 25 [13]	**This Work**
Application	Commercial high-voltage DAC	Optical phased control	OLED driver	OLED driver	**Optical phased control**
Process	N/A	130 nm CMOS	180 nm CMOS	65 nm CMOS	**250 nm** **CMOS**
DAC resolution (bit)	14	8	10	10	**8**
Output range (V)	−4 to 8	N/A	0.2 to 4.8	0.2 to 4.8	**0.2 to 8.2**
Number of channels	40	2048	40	40	**128**
Max DNL/INL (LSB)	1/1	0.5/0.5	0.46/0.89	0.42/1.44	**0.26/0.39**
Max DVO (mV)	0.7	N/A	7.5	14.3	**22.3**
Max DVO (LSB)	1	N/A	1.66	3.18	**0.71**
Calibration method	Gain/offset calibration	N/A	Offset calibration	N/A	**Per-code calibration**
Calibration memory	On-chip trim registers	N/A	N/A	N/A	**16 kB external LUT**
Power/ch. (mW)	7	0.125	N/A	N/A	**6.64**
Area/ch. (μm^2^)	N/A	6241	6444	1884	**53,750**
Load condition	10 kΩ, 200 pF	N/A	30 kΩ, 30 pF	N/A	10 kΩ, 5 pF
Slew rate rise/fall (V/μs)	1	N/A	N/A	4.6/3.2	**6.1/11.7**

## Data Availability

Data underlying the results presented in this paper are not publicly available at this time but may be obtained from the authors upon reasonable request.
